# Screening for *K13-*Propeller Mutations Associated with Artemisinin Resistance in *Plasmodium falciparum* in Yambio County (Western Equatoria State, South Sudan)

**DOI:** 10.4269/ajtmh.23-0382

**Published:** 2023-09-25

**Authors:** Irene Molina-de la Fuente, María José Sagrado Benito, Janet Ousley, Francisco de Bartolomé Gisbert, Luz García, Vicenta González, Agustín Benito, Buai Tut Chol, Ahmed Julla, Abubakr Bakri, Carolina Nanclares, Pedro Berzosa

**Affiliations:** ^1^ Department of Biomedicine and Biotechnology, School of Pharmacy, University of Alcalá, Alcalá de Henares, Madrid, Spain;; ^2^ Malaria and Neglected Diseases Laboratory, National Centre of Tropical Medicine, Institute of Health Carlos III, Madrid, Spain;; ^3^ CIBERINFECT – CIBER Infectious Diseases, Madrid, Spain;; ^4^ Médecins Sans Frontières, Barcelona, Spain;; ^5^ Médecins Sans Frontières, New York, New York;; ^6^ Médecins Sans Frontières, Juba, South Sudan;; ^7^ Ministry of Health, Juba, South Sudan;; ^8^ Médecins Sans Frontières, Nairobi, Kenya

## Abstract

Artemisinin-combined treatments are the recommended first-line treatment of *Plasmodium falciparum* malaria, but they are being threatened by emerging artemisinin resistance. Mutations in *pfk13* are the principal molecular marker for artemisinin resistance. This study characterizes the presence of mutations in *pfk13* in *P. falciparum* in Western Equatoria State, South Sudan. We analyzed 468 samples from patients with symptomatic malaria and found 15 mutations (8 nonsynonymous and 7 synonymous). Each mutation appeared only once, and none were validated or candidate markers of artemisinin resistance. However, some mutations were in the same or following position of validated and candidate resistance markers, suggesting instability of the gene that could lead to resistance. The R561L nonsynonymous mutation was found in the same position as the R561H validated mutation. Moreover, the A578S mutation, which is widespread in Africa, was also reported in this study. We found a high diversity of other *pfk13* mutations in low frequency. Therefore, routine molecular surveillance of resistance markers is highly recommended to promptly detect the emergence of resistance-related mutations and to limit their spread.

## INTRODUCTION

Malaria is a serious disease that continues to pose a global health challenge, particularly in Africa. In 2021, there were an estimated 247 million malaria cases globally, of which 95% were located in African countries.^1^ One of the key malaria control strategies in endemic areas is artemisinin-based combination therapy (ACT), which includes an artemisinin derivative with another antimalarial drug. Artemisinin-based combination therapy is adopted worldwide as the first-line treatment against uncomplicated falciparum malaria.^1^ It has demonstrated good efficacy even against parasites resistant to other antimalarials.^2^

Artemisinin-based combination therapy has successfully contributed to the reduction of the global burden of malaria; however, the emergence and spread of artemisinin resistance threatens its efficacy.^1^ Artemisinin partial resistance, characterized by delayed parasite clearance after treatment, is widespread in the Greater Mekong subregion in Southeast Asia, and it has been generally absent in Africa.^3^ However, the WHO recently confirmed the presence of artemisinin partial resistance in Rwanda, Uganda, and Eritrea.^4^^,^^5^

Artemisinin partial resistance has been associated with mutations in the propeller domain of the gene *Pfk13,* encoding the Kelch 13 (K13) protein, which are associated with resistant phenotypes in vitro and in vivo.^6^ So far, the WHO has recognized 13 *pfk13* nonsynonymous mutations (i.e., resulting in a change of amino acid) as validated molecular markers of resistance and 9 as candidate markers of resistance.^3^ These mutations are located in the BTB/POZ domain and the C-terminal six-blade propeller regions of the pfK13 protein.^6^

The majority of the *pfK13* mutations that have been identified in Africa are not validated resistance markers.^7^^–^^9^ However, some of the *pfK13* validated mutations and their clonal expansion have been reported recently in *Plasmodium falciparum* parasites in Africa.^10^ These mutations have been found in different frequencies in Rwanda,^11^ Tanzania,^12^ Uganda,^13^ and Ethiopia^14^ (the latter two sharing a border with South Sudan). Although Rwanda and Uganda have both confirmed the presence of partial artemisinin resistance, ACTs continue to be effective, and treatment failure rates remain below 10% in these countries.^10^^–^^15^ The WHO African Region has declared surveillance of *pfk13* polymorphisms a priority for malaria control.^1^

Malaria is a major public health problem in South Sudan, where it is the leading cause of illness and death.^16^ Over 93% of malaria cases in the country are caused by *P. falciparum*,^16^ and malaria incidence and mortality rates are on the rise.^1^ The government health authorities adopted artesunate-amodiaquine as the first-line antimalarial treatment in 2006.^17^

Given increasing malaria incidence and mortality in South Sudan and the emergence of artemisinin resistance in other countries in east Africa, studies on molecular markers are highly recommended in this country.^1^

We aimed to investigate the presence of *pfk13* gene mutations related to artemisinin resistance in the malaria-endemic region of Yambio County (Western Equatoria State, South Sudan) 14 years after the introduction of ACT in the country.

## MATERIALS AND METHODS

### Study area and biological samples.

In this retrospective analysis, we assessed samples that had been collected for a study led by Médecins Sans Frontières in collaboration with the South Sudanese Ministry of Health to investigate molecular markers of antimalarial drug resistance after implementation of a seasonal malaria chemoprophylaxis campaign in Western Equatoria State, South Sudan. The study was conducted in a rural, malaria-endemic area in Yambio County at the end of the malaria peak season (from January to February 2019).

**Figure 1.**
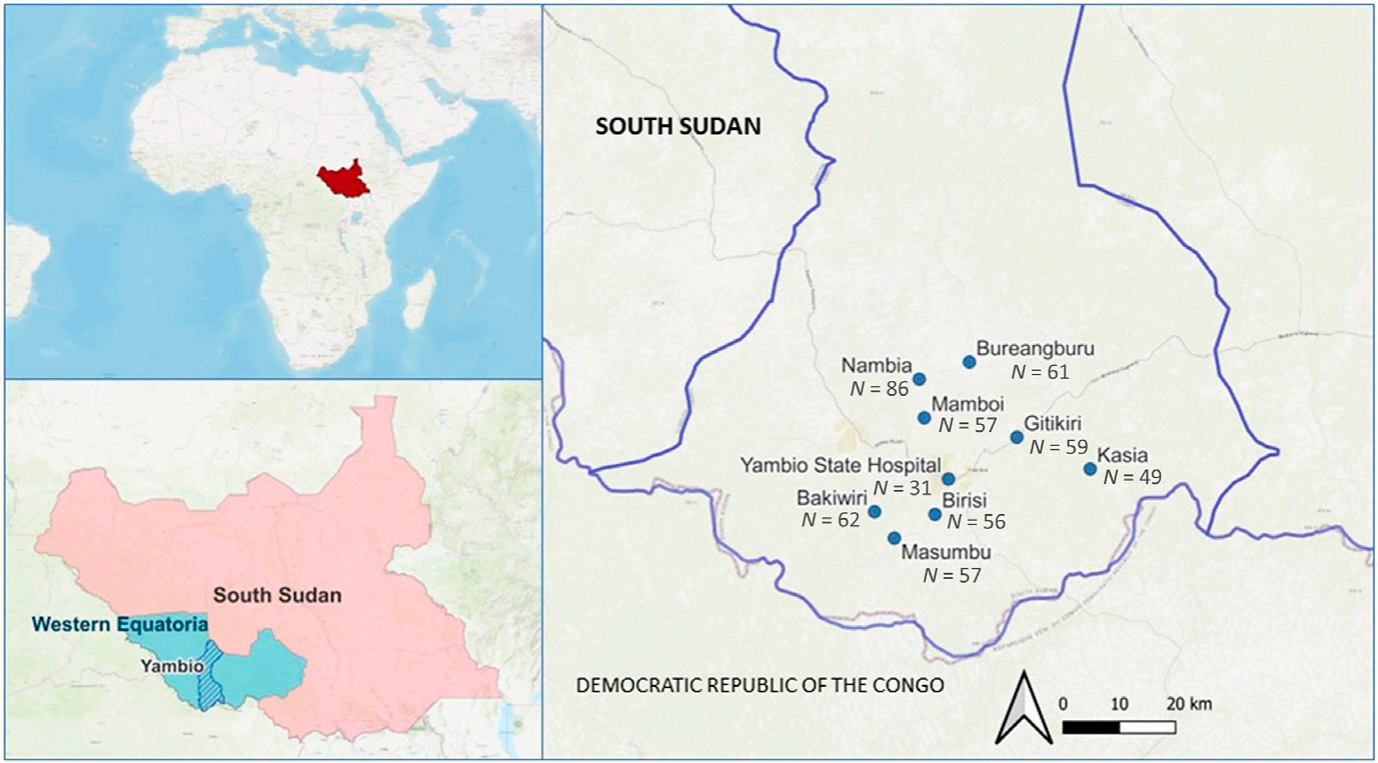
Map of the sample collection sites in Yambio County, Western Equatoria State, South Sudan. *N* = sample size per collection site.

Finger-prick, dried blood spot (DBS) samples were collected using Whatman 903 paper (GE HealthCare Bio-Sciences Corp., Chicago, IL) from the general population aged > 6 months who were presenting symptoms compatible with malaria and for whom malaria infection was confirmed by pan lactate dehydrogenase-based-rapid diagnostic test in eight community-based treatment sites and in the referral hospital (Yambio State Hospital) (Figure 1). Most samples were from patients with uncomplicated malaria, except for the samples from the Yambio State Hospital, which were from patients with severe malaria. The DBS samples were sent for analysis to the National Center of Tropical Medicine (Madrid, Spain) following standard procedures.

### DNA extraction.

We extracted DNA from the DBS samples using the Saponin/Chelex method with minor modifications from our laboratory.^18^ We used a punch of 5-mm diameter containing 10 μL of blood. The tube containing the isolated DNA was labeled with the sample number and year and place of collection. These DNA samples were either used immediately for polymerase chain reaction (PCR) or stored at −20°C until further use.

### Screening for *Pfk13* mutations.

We used nested multiplex PCR to confirm *P. falciparum* diagnosis for all samples.^18^^,^^19^ Then, we amplified the *pfk13* gene using nested PCR, as described by Ariey et al.,^6^ with minor modifications from our laboratory including the use of another Polymerase HotStart (5 U/mL) (Biotools, Madrid, Spain) and lower annealing temperature (58°C) and MgCl_2_ concentration (2 mM) for primary PCR. We verified the size of the amplification fragment (±850 bp) by electrophoresis on 2% agarose gel stained with Pronasafe (Pronadisa, Spain).

Next, we purified the PCR products using Illustra ExoProStar 1-step (GE Healthcare Life Sciences, Marlborough, MA) according to the manufacturer’s instructions and sequenced these products from both directions by a standard dye terminator Big Dye Terminator v3.1 Cycle Sequencing kit (Thermo Fisher Scientific, Madrid, Spain) in an ABI PRISM 3730 XL Analyser (Thermo Fisher Scientific). We checked these sequences with BLAST and compared them against a *pfk13* 3D7 clone using BioEdit 7.2 software to find new and validated mutations.

### Ethics.

This study was approved by the internal ethics review board at Médecins Sans Frontières and by the South Sudan Research Ethics Committee. All participants or children’s parents or guardians provided written informed consent.

## RESULTS

Of 601 samples analyzed, 518 had a PCR-confirmed *P. falciparum* mono-infection. *Pfk13* was successfully amplified and sequenced in 468 of these samples. Amplification of *Pfk13* was unsuccessful for 50 samples.

We detected 15 different mutations in 14 (3%) of the 468 samples (one sample had two mutations). Eight of these 15 mutations were nonsynonymous (53%), and seven were synonymous (47%). None of the eight nonsynonymous mutations were validated or candidate mutations associated with artemisinin resistance listed by the WHO.

The distribution of mutations in the propeller domain of *pfK13* revealed that the majority of mutations were in blades 2 (*n =* 4) and 4 (*n =* 4). The rest of the blades (blades 3, 5, and 6) had two mutations each, except for blade 1 (*n =* 1) and BTB/POZ (*n =* 0).

All geographical locations presented at least one parasite with a mutation in the *PfK13* gene (Table 1), with Yambio Hospital being the site with the highest frequency of mutations (8%, 2/26). The R561L nonsynonymous mutation was found in the same position as the R561H validated mutation by the WHO and the S623G nonsynonymous mutation in the following position to the R622I validated mutation. In addition, three synonymous mutations were observed either in the same position or adjacent to a candidate mutation point for resistance (Table 2).

**Table 1:** Frequency of *pfk13* synonymous and nonsynonymous mutations by location

Location	*N*	Codon	Wild allele	AA	Mutant allele	AA	Mutation	Type	Frequency *n* (%)	Previous identification (WHO region)
			Sequence (nt)		Sequence (nt)					
Nambia	86	623	AGT	S	GTT	G	S623G	NS	4 (4.7%)	Africa*
		654	CCA	Q	CAG	Q	Q654Q	S		–
		519	TAT	Y	CAT	H	Y519H	NS		None (novel)
		561	CGT	R	CTT	L	R561L	NS		Africa*
Momboi	55	605	GAA	E	AAA	K	E605K	NS	1 (1.8%)	None (novel)
Gitikiri	50	486	GCT	A	TCT	S	A486S	NS	1 (2.0%)	None (novel)
Bakiwiri	57	694	AAT	N	AAC	N	N694N	S	2 (3.5%)	–
		516	GAT	D	GAC	D	D516D	S		–
Yambio State Hospital	26	673	TTT	F	TTC	F	F673F	S	2 (7.7%)	Africa†
		578	GCT	A	TCT	S	A578S	NS		Africa
Masumbu	50	567	GAG	E	GAA	E	E567E	S	1 (2.0%)	–
Birisi	50	602	GAA	E	AAA	K	E602K	NS	1 (2.0%)	None (novel)
Bureangburu	51	496	GGT	G	GGC	G	G496G	S	3 (5.9%)	Africa†
		615	CCA	P	TCA	S	P615S	NS		Africa*
		449	GGT	G	GGC	G	G449G	S		Africa†
Kasia	43	–	–	–	–	–	–	–	0	–

AA = amino acid; NS = not significant; nt = nucleotide; S = significant.

* Reported mutation with other change.

† Reported NS mutation.

**Table 2:** Relatedness between mutations found in South Sudan and mutations associated with resistance to artemisinin

Validated mutations by the WHO	Candidate mutations by the WHO	Potentially associated mutations*	Mutations South Sudan	Location
R561H	–	–	R561L	Nambia
R622I	–	–	S623G	Nambia
–	G449A	–	G449G	Bureangburu
–	V568G	–	E567E	Musumbu
–	R515K	–	D516D	Bakiwiri
–	–	M579I	A578S	Yambio State Hospital
–	–	F673I	F673F	Yambio State Hospital

* These mutations have been associated with partial resistance; however, because of small sample size, the associations were statistically nonsignificant.^20^

## DISCUSSION

This study reveals the presence of 15 mutations, 8 nonsynonymous and 7 synonymous, in *pfK13* in South Sudan, some of which are novel. Similar to findings in other sub-Saharan countries,^21^^,^^22^ none of these mutations were in the WHO list of resistance-associated markers. However, any new mutations detected in *pfk13* could potentially be a new indicator of drug resistance development and should be reported.^23^^,^^24^ Moreover, the majority of mutations detected were in blades 2 and 4, which are responsible for protein structure.^20^^,^^25^ Although knowledge is still limited, it seems that changes in these blades could alter the integrity of the protein and potentially lead to resistance.^6^^,^^25^^,^^26^

Recent reports of emerged mutations in Africa highlight the crucial need for surveillance to limit their spread and try to avoid potential consequences on ACT efficacy.^24^ Recently, northern Uganda, bordering South Sudan, reported a high presence of molecular markers related to artemisinin partial resistance, including an increasing trend in frequencies for C469Y and A675V, both associated with delayed parasite clearance.^13^^,^^15^ Although these mutations have not been found in South Sudan, there is a risk of introduction and spread to this country.

The A578S mutation, a widespread *pfK13* mutation in sub-Saharan Africa including Uganda, Kenya, and Republic of the Congo, was detected in our study.^9^ This mutation has not been associated with reduced susceptibility to artemisinin,^26^^,^^27^ although one report from Uganda has linked it with possible artemisinin resistance.^28^ For this reason and because of its extensive distribution and its position near the A580Y validated resistance mutation,^29^ it would be relevant to continue surveillance to monitor its impact on parasite clearance.

None of the mutations detected in South Sudan correspond to validated or candidate artemisinin-resistance mutations; however, some are either in the same or in an adjacent position as other validated or candidate mutations previously reported elsewhere in Africa. Although there are no data on the biological significance of these mutations, these changes could precede validated mutations or could potentially affect susceptibility to artemisinin.

For example, the nonsynonymous mutation R561L was detected in the same position as the WHO validated R561H resistance marker, which has been found to be present in Rwanda.^10^ These variants with mutations in the same position as a validated mutation but with a change to a different amino acid need to be carefully monitored and assessed, as they could potentially affect antimalarial activity of artemisinins.^26^

The synonymous mutation G449G is in the same position as the WHO candidate resistance mutation G449A, and the presence of various mutations (e.g., S623G, A578S) in positions adjacent to those of validated or candidate resistance markers should be highlighted, as they indicate instability in these specific areas of the gene.^3^^,^^30^^,^^31^

In addition, we detected the E605K mutation, which was also reported in Ghana.^23^ This mutation has not been associated with resistance, but it could correspond to an unstable position where mutations occur more easily, albeit without a known biological effect.

Recent reports, including our findings, on the emergence in Africa of spontaneous *Pfk13* mutations that can be associated with delayed clearance or even resistance suggest that continued molecular and clinical monitoring is crucial, especially in areas of high transmission such as South Sudan. A high rate of recombination, associated with high transmission, increases the risk of new mutations, although selection may be suppressed by high diversity.^23^ Some authors suggest that the mutations described in Africa have an independent origin from the ones in Asia, conforming different genetic clusters, so it is expected that more resistance-associated mutations may emerge independently in Africa.^8^ Moreover, a diversifying selection of parasites with mutations in *pfk13*, likely due to adaptation of the parasite to ACTs, could possibly endanger the antimalarial activity of these medications, threatening malaria control.^32^^,^^33^ In the case of Yambio County (South Sudan), we found a wide variety of mutations but in low frequency.

This study has some limitations. It is important to highlight that these samples were collected in 2020; hence, the scenario may have changed, especially considering rapid spread in neighboring Uganda. Results cannot be extrapolated to the rest of the country as samples were collected from only one state and areas farther east bordering Uganda could potentially have a higher prevalence of mutations. Moreover, as therapeutic efficacy was not evaluated in this study, it is not possible to determine the impact of the *pfk13* mutations reported on parasite clearance half-life.

## CONCLUSIONS

We found no validated or candidate markers associated with artemisinin resistance in Yambio County, Western Equatoria State (South Sudan). However, we reported a high diversity of other *Pfk13* mutations in low frequency. Some of these mutations were either in the same positions or in adjacent positions as validated or candidate markers of resistance, suggesting instability of the gene that could lead to resistance. Therefore, routine molecular surveillance of resistance markers is highly recommended to promptly detect the emergence of resistance-related mutations and to limit their spread. In addition, routine monitoring of antimalarial drug efficacy is needed.^1^
